# Effects of Mn Deficiency on Hepatic Oxidative Stress, Lipid Metabolism, Inflammatory Response, and Transcriptomic Profile in Mice

**DOI:** 10.3390/nu17193030

**Published:** 2025-09-23

**Authors:** Yaodong Hu, Shi Tang, Silu Wang, Caiyun Sun, Binlong Chen, Binjian Cai, Heng Yin

**Affiliations:** 1 College of Animal Science, Xichang University, Xichang 615000, China; tangshi1989103@163.com (S.T.); 13_wsl@163.com (S.W.); suncaiyun1237@gmail.com (C.S.); binlong2369@163.com (B.C.); cai050806@126.com (B.C.); 2 College of Life Sciences and Agri-Forestry, Southwest University of Science and Technology, Mianyang 621010, China; yinheng0123@163.com

**Keywords:** Mn deficiency, mouse, liver, PPAR

## Abstract

**Introduction:** Mn is a trace element essential for growth and development in organisms, and adequate Mn levels are crucial for maintaining normal liver function. This study aimed to investigate the effects of Mn deficiency on the liver and elucidate the underlying mechanisms using transcriptomics. **Methods:** Weanling mice were fed a Mn-deficient diet, and Mn chloride (MnCl_2_) was administered intraperitoneally to correct the deficiency. Liver pathological changes were evaluated through histological examination. Liver function and key lipid metabolism markers were assessed using biochemical assays, while hepatic oxidative stress levels were measured via flow cytometry and biochemical kits. Alterations in inflammatory factors were detected using ELISA and qPCR. The mechanisms underlying Mn’s effects on liver function were further explored through Western blot, qPCR, and transcriptome sequencing. **Results:** Mn deficiency impaired liver morphology and structure. Serum levels of ALT, AST, and ALP were significantly elevated, while ALB decreased, confirming hepatic dysfunction. This dysfunction led to oxidative stress, characterized by increased hepatic ROS and MDA levels, alongside reduced Mn-SOD, GSH-Px, and T-AOC activities. Additionally, Mn deficiency elevated serum TG, TC, and LDL-C levels, indicating abnormal lipid metabolism. Hepatic pro-inflammatory factors (IL-6, IL-1β, and TNF-α) were significantly upregulated. Transcriptomic analysis revealed distinct gene expression patterns under different Mn conditions, with KEGG pathway analysis identifying the PPAR signaling pathway as a key regulatory target. **Conclusions:** Our findings suggest a potential pathogenic cascade in which manganese deficiency may initially induce hepatic oxidative stress, potentially leading to suppression of the PPAR signaling pathway. This inhibition of PPARα/γ could subsequently orchestrate downstream manifestations of aberrant lipid metabolism and inflammatory responses. Thus, the PPAR signaling pathway is proposed as a plausible central hub for translating oxidative damage into metabolic and inflammatory dysfunction in the manganese-deficient liver.

## 1. Introduction

Manganese (Mn) is an essential trace element primarily acquired through dietary sources such as nuts, whole grains, and leafy vegetables, as well as through drinking water [1]. The homeostasis of manganese in the body is regulated through specific transport pro-teins, such as divalent metal transporter 1 (DMT1) and SLC39A8 (ZIP8) [2,3]. Following intestinal absorption, dietary manganese is predominantly bound to γ-globulin and albumin for systemic circulation in the blood. Under physiological conditions, manga-nese accumulates at relatively high concentrations in metabolically active tissues, in-cluding the liver, pancreas, and pituitary gland [4]. The liver plays a dominant role in systemic Mn regulation, as it not only stores Mn but also excretes excess amounts through bile [5,6]. Mn serves as a critical cofactor for enzymes involved in diverse biological processes, including bone formation, cholesterol and carbohydrate metabolism, and neurotransmitter synthesis [7,8]. Notably, it is an indispensable component of Mn superoxide dismutase (Mn-SOD), the primary antioxidant enzyme in mitochondria responsible for neutralizing superoxide radicals (O_2_•^−^) and protecting against oxidative damage [9]. Under physiological conditions, Mn homeostasis is tightly regulated, but deficiency can arise from inadequate intake, genetic defects in Mn transporters, or competition with other metals like iron. Experimental studies have demonstrated that Mn deficiency leads to growth retardation, skeletal deformities, impaired glucose metabolism, and dyslipidemia [10]. According to the European Food Safety Authority (EFSA), the safe daily intake of manganese for adults aged 18 and above—including pregnant and lactating women—is established at 8 milligrams per day. For other demographic groups, the recommended intake ranges from 2 to 7 milligrams per day [11].Under normal conditions, humans can acquire sufficient manganese through diet, making dietary manganese deficiency relatively uncommon [12]. Notably, animal-based foods such as red meat, seafood, and poultry contain very low manganese, leaving plant-based foods as the primary source [13]. With increasing social industrialization, traditional plant-based diets are being replaced by Western-style diets rich in red meat, high-sugar desserts, high-fat foods, and refined grains. This shift may lead to reduced manganese intake in the population [14]. Epidemiological data show a 42.4% decline in manganese intake in the U.S. over 15 years, though one study focused on high-fiber consumers and the other was a total diet study [15,16]. Similarly, in Korea, adults’ daily manganese intake fell from 5.21 mg (2008) [17] to 1.8 mg (2014) [18], highlighting a clear downward trend and the need to understand physiological impacts of manganese deficiency.

The liver serves as the metabolic hub of the human body, with Mn playing a vital role in hepatic health due to its involvement in numerous metabolic processes. Consequently, Mn imbalance—whether deficiency or excess—is strongly linked to the development and progression of metabolic liver disorders [19,20,21]. Research demonstrates that Mn is essential for maintaining hepatic structural integrity, and its deficiency leads to significant structural and functional impairments in liver tissue [22]. Evidence suggests a U-shaped dose–response relationship between Mn levels, reactive oxygen species (ROS) production, and oxidative stress, wherein both deficiency and excess disrupt redox homeostasis and exacerbate oxidative damage.

This disruption in redox balance, particularly under Mn deficiency (MnD), has profound consequences. While ROS generation is crucial for immune responses—promoting leukocyte activation, proliferation, and cytokine secretion—excessive ROS impairs immune cell function, potentially leading to immune tolerance, hyperactivation, and subsequent autoimmune or chronic inflammatory conditions [23]. Mn deficiency may further aggravate inflammation by disrupting immune homeostasis. Under conditions of Mn deficiency (MnD), excessive ROS production can activate pro-inflammatory signaling pathways, such as NF-κB and the NLRP3 inflammasome, upregulating cytokines like TNF-α and IL-6 [24]. This chronic low-grade inflammation is a hallmark of metabolic liver diseases [25]. Importantly, the hepatic oxidative stress and inflammation triggered by MnD directly drive metabolic dysfunction within the liver. Experimental studies indicate that MnD reduces Mn-SOD activity, further promoting hepatic oxidative stress [26]. Concurrently, this oxidative stress and inflammation initiate a vicious cycle of dysregulated lipid metabolism: Oxidative stress damages hepatocytes via free radicals, suppresses fatty acid β-oxidation, and enhances lipid synthesis, leading to lipid accumulation [27,28]. Conversely, accumulated lipids (e.g., free fatty acids) induce mitochondrial dysfunction and excessive ROS production, further aggravating oxidative stress, inflammation, and liver injury [29]. This cycle ultimately manifests as hepatic steatosis and insulin resistance, core features of disorders like non-alcoholic fatty liver disease (NAFLD).

However, existing research into the effect of Mn deficiency upon the liver is scarce. Hence, the purpose of the research is to investigate the impact of Mn deficiency on liver function by inducing Mn deficiency through dietary restriction in mice. It seeks to assess alterations of the Lipid Dyshomeostasis and oxidative stress level in the liver, so as to uncover the potential mechanisms underlying these effects.

## 2. Materials and Methods

### 2.1. Reagents

MnCl_2_ (cat no 13446-34-9AR 99%) came from Chengdu Kelong Chemical Co., Ltd. (Chengdu, China). The kits for ascertaining biochemical parameters, such as aspartate aminotransferase (AST; cat no. C010-2-1), alanine aminotransferase (ALT; cat no. C009-2-1), albumin (ALB; cat no. A028-2-1), alkaline phosphatase (ALP; cat no. A059-2-2), triglyceride (TG; cat no. A110-1-1), low-density lipoprotein cholesterol (LDL-C; cat no. A113-1-1), total cholesterol (TC; cat no. A111-1-1), high-density lipoprotein cholesterol (HDL-C; cat no. A112-1-1), superoxide dismutase (SOD; cat no. A001-2-2), total antioxidant capacity, (T-AOC; cat no. A015-3-1), glutathione peroxidase (GSH-px; cat no. A005-1-2)and malondialdehyde (MDA; cat no. A003–1) were purchased from Nanjing Jiancheng Bioengineering Institute of China (Nanjing, China). The ELISA Kits of IL-6 (cat no. EMC004QT.96), IL-1β (cat no. EMC001B.96), and TNF-α (cat no. 102EMC102a.48) came from Neobioscience. The following antibodies were involved in this study: anti-rabbit PPARγ (Cat.# 2435 CST), anti-rabbit PPARα (ab314112 Abcam, Cambridge, UK), anti-rabbit CD36 (18836-1-AP Proteintech, Wuhan, China), anti-rabbit FABP4 (12802-1-AP Proteintech), and β-actin (EM21002 HUABIO, Hangzhou, China).

### 2.2. Animals and Treatments

Forty-five 3-week-old male ICR mice were obtained from Dashuo Biological Technology Co. (Chengdu, China) and acclimatized in a pathogen-free animal facility under controlled conditions (25 ± 2 °C, 55 ± 5% humidity). Following the acclimatization period, The animals were randomly divided into three experimental groups: (1) control (standard diet + saline), (2) Mn-deficient (MnD, Mn-deficient diet + saline), and (3) MnD + MnCl_2_ (Mn-deficient diet + MnCl_2_ supplementation). Randomization was performed using a computer-generated random number sequence (Microsoft Excel) by an independent researcher to avoid selection bias. The sample size was determined using G*Power software (version 3.1.9.7). In accordance with Cohen’s conventional thresholds for effect sizes in analysis of variance, a conservatively large effect size (f = 0.4) was selected for power calculation. The results indicated that, at a significance level (α) of 0.05 and with 80% statistical power, 22 mice per group would be required. However, in alignment with the Reduction principle of the 3R guidelines and taking into account common sample sizes employed in comparable studies) [30,31], the final sample size was set at 15 mice per group.All mice had ad libitum access to their respective experimental diets and water. Mice were group-housed (5 mice per cage) in standard ventilated cages.Bedding was changed every two days. For four weeks, the control group received a standard pellet diet, while the MnD and MnD + MnCl_2_ groups were fed a Mn-depleted diet. During the final week, the control and MnD groups were administered saline, whereas the MnD + MnCl_2_ group received intraperitoneal MnCl_2_ (4 mg/kg, based on Lee et al. [32]). On the final day of the experiment, The mice were randomly selected from each group, weighed, and anesthetized with isoflurane. Blood was collected from the eyeballs and placed in clean 1.5 mL centrifuge tubes for standing. After blood collection, euthanasia was performed by cervical dislocation. The purified diets, formulated in accordance with the AIN-76A rodent maintenance guidelines (American Institute of Nutrition), were supplied by SPF Biotechnology Co. (Beijing, China). Nutritional composition, including Mn levels (MnD diet: 0–3 ppm), is detailed in Table 1. All procedures complied with the National Research Council’s guidelines for laboratory animal welfare and were approved by the Institutional Animal Care and Use Committee of Southwestern University of Science and Technology (approval code LX202300521). In this study, all dietary interventions represented standard feeding practices and were not associated with pain or distress in the animals. Throughout the experimental period, all animals were closely monitored on a daily basis for health status and behavioral performance. Dedicated animal welfare officers conducted systematic assessments of animal appearance (e.g., coat condition), behavior (e.g., activity level), and physiological parameters (e.g., body weight changes) to evaluate potential signs of pain or suffering. Clear humane endpoint criteria were established prior to the study. Should any animal have exhibited severe distress—such as weight loss exceeding 20%, inability to access food or water independently, or profound weakness—prompt intervention, including euthanasia, would have been implemented. Notably, no animals displayed any signs of pain, distress, or discomfort necessitating additional intervention during the course of the study.

### 2.3. Histopathological Observation

After being collected, the livers of six mice in each group were fixed with 4% paraformaldehyde. After fixation, dehydration with gradient ethanol and paraffin-embedding, the samples were sliced into sections with a thickness of 4 μm and hematoxylin and eosin (H&E) stained. A light microscope was adopted to observe histopathological changes.

### 2.4. Transcriptome Sequencing and Transcriptomic Analysis

The samples were collected for transcriptome sequencing. A Ribo-Zero Magnetic Kit was used to remove rRNA and obtain RNA. Under the action of reverse transcriptase, first-strand cDNA was synthesized using random primers and mRNA as templates. During the second-stand synthesis, dTT’P was replaced by dUT’P in thedN’T’Ps reagent. After quantification with TBS380 (Picogreen, Eugene, OR, USA), RNA-seg double-end sequencing was performed using an lllumina HiSeg X Ten (2 × 150 bp), Illumina, San Diego, CA, USA. Bioinformatic analysis was performed using data generated by the lllumina platform. The RNA-Seq FASTQ files were aligned with the mouse genome (ensembl mus musculus_grcm38_p6_gca 000001635 8) using the Hisat2 algorithm. Allanalyses were performed using the cloud platform (www.majorbio.com, accessed on 16 May 2025) fom Majorbio Co., Ltd., Shanghai, China.

### 2.5. Liver ROS Assessment

Take 50 mg of fresh liver tissue and homogenize it according to the ROS kit instructions (Bestbio, Shanghai, China). Centrifuge at 100× *g*, 4 °C for 3 min, then take 200 μL of the supernatant. Add 2 μL of the probe, mix well, and incubate at 37 °C for 30 min. Take a portion of the supernatant for protein content determination, and then perform ROS detection using flow cytometry.

### 2.6. Oxidative Stress Assessment in the Liver

After weighing, liver tissues were homogenized using normal saline with nine times the amount thereof, and then centrifuged at 3500 rpm and 4 °C for 10 min, followed by collecting the supernatant. Subsequently, the biochemical method was selected to test the MDA content, CAT content, GSH-Px content, Mn-SOD activity, and the total T-AOC activity using biochemical reagent kits obtained from Nanjing Jiancheng Bioengineering Institute of China (Nanjing, China).

### 2.7. Quantitative Real-Time PCR (qRT-PCR) Analysis

An RNA kit (Tianmo Biotech, Beijing, China) was used to extract the total RNA. It was then transformed into cDNA through use of 5X RT Mix (Beijing Biomed Gene Technology Co., Ltd., Beijing, China). Quantitative fluorescence detection using EvaGreen 2XqPCR MasterMix (AbM, Vancouver, BC, Canada). Primer 5.0 (Table 2) was used to design all primer sequences. The 2^−∆∆CT^ method was utilized to compute the target transcript level relative to β-actin.

### 2.8. Western Blotting Analysis

Liver tissue homogenates were prepared using RIPA buffer, and total protein levels were quantified with a BCA assay. Protein separation was carried out via SDS-PAGE, followed by electrophoretic transfer onto PVDF membranes. The membranes were then blocked with 5% non-fat milk for 1 h. Primary antibodies targeting PPARα, PPARγ, CD36, and FABP4 were applied and incubated overnight at 4 °C. After washing, horseradish peroxidase (HRP)-conjugated secondary antibodies were introduced and incubated for an additional hour. Protein bands were visualized using a ChemiDoc XRS imaging system with an ECL substrate, and densitometric analysis was performed using ImageJ1.8.0 software.

### 2.9. Statistical Analysis

Data expressed as means ± standard deviations were subjected to variance analyses (LSD or Dunnett’s T3). SPSS software, version 22.0 (IBM Corp., Armonk, NY, USA) for windows was utilized to perform all statistical analyses. *p* < 0.05 suggested a significant difference.

## 3. Results

### 3.1. Effects of Mn Deficiency on Hepatic Histopathology and Liver Function in Mice

**Figure 1:**
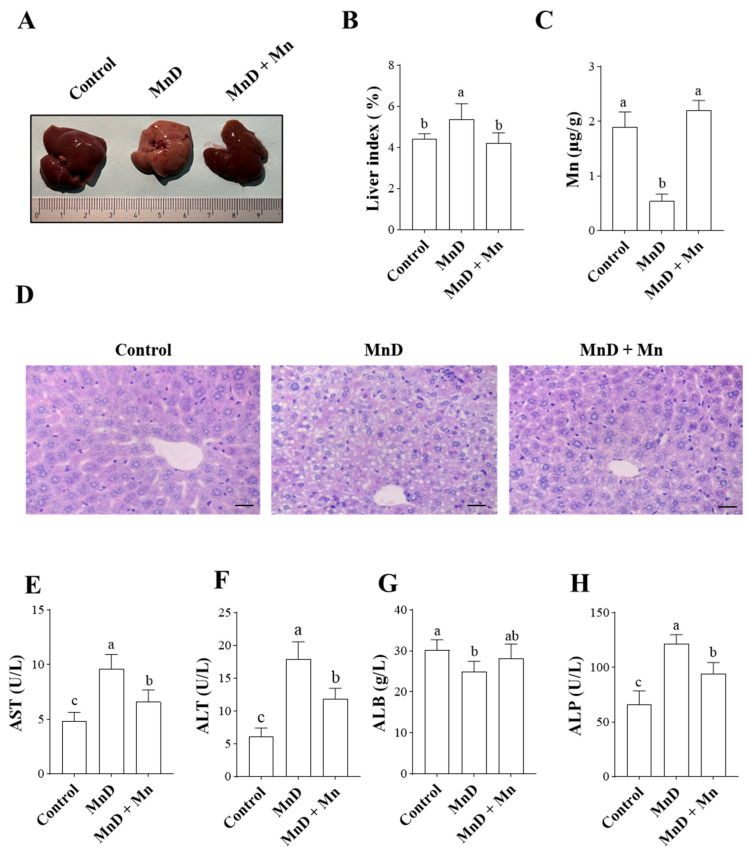
Effects of Mn Deficiency on Hepatic Histopathology and Liver Function in Mice. (**A**) liver macrostructure; (**B**) liver index; (**C**) the concentrations of Mn in liver; (**D**) the histopathological changes in the liver (HE staining, scale bar  =  50 µm); (**E**–**H**) the concentrations of AST, ALT, ALB, and ALP in liver. Data are presented with the mean  ±  standard deviation (n  =  6). ^abc^ Different letters represent significant difference (*p*  <  0.05) within the column, and the same letters represent no significant difference (*p*  >  0.05).

The morphological characteristics, organ index, Mn content, and histological structure of mouse livers are presented in Figure 1A–D. As shown in Figure 1A, livers from the Mn-deficient (MnD) group exhibited significantly paler coloration and oily surface compared to other groups. Figure 1B demonstrates that the liver index was significantly elevated (*p* < 0.05) in the MnD group relative to controls, whereas Mn supplementation (MnD + MnCl_2_) restored this parameter to near-normal levels. Hepatic Mn content (Figure 1C) was markedly reduced (*p* < 0.05) in MnD mice compared to controls, but normalized following MnCl_2_ treatment. Histopathological examination (Figure 1D) revealed distinct differences between groups: control livers displayed normal hepatic cord arrangement and hepatocyte morphology, while MnD livers exhibited structural abnormalities including disorganized hepatic cords, extensive hepatocyte steatosis, and narrowed hepatic sinusoids. These pathological changes were substantially ameliorated in the MnD + MnCl_2_ group. Additionally, we assessed established liver injury biomarkers (AST, ALT, ALB, and ALP). As illustrated in Figure 1E–H, serum AST, ALT, and ALP levels were significantly elevated in the MnD group (*p* < 0.05), whereas ALB was significantly reduced (*p* < 0.05) compared to controls. Mn supplementation partially or completely reversed these alterations.

### 3.2. Effects of Mn Deficiency on Oxidative Stress in the Mice

**Figure 2:**
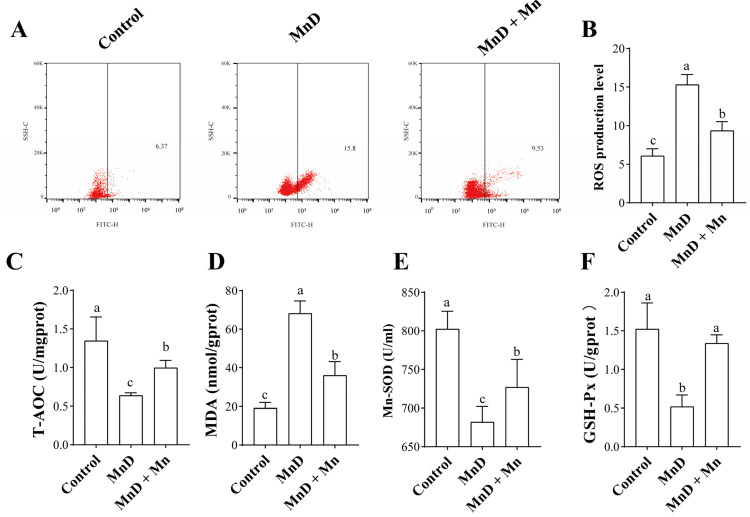
Effects of Mn deficiency on oxidative stress in the mice. (**A**,**B**) Relative ROS amounts determined by flow cytometry, (**C**) T-AOC; (**D**) MDA; (**E**) Mn-SOD; (**F**) GSH-Px. Data are presented with the mean  ±  standard deviation (n  =  6). ^abc^ Different letters represent significant difference (*p*  <  0.05) within the column, and the same letters represent no significant difference (*p*  >  0.05).

Figure 2 demonstrates the impact of Mn deficiency and MnCl_2_ supplementation on hepatic oxidative stress in mice. As shown in Figure 2A,B, hepatic reactive oxygen species (ROS) levels were significantly elevated (*p* < 0.05) in the Mn-deficient (MnD) group compared to controls. While MnCl_2_ supplementation (MnD + MnCl_2_ group) significantly reduced ROS content relative to the MnD group, levels remained higher than in controls (*p* < 0.05). Figure 2C reveals that malondialdehyde (MDA) concentrations were markedly increased in the MnD group compared to controls, with MnCl_2_ treatment significantly lowering MDA levels, though these remained elevated above control values. Figure 2D–F demonstrate that Mn deficiency significantly decreased both the content or activity of total antioxidant capacity (T-AOC), Mn superoxide dismutase (Mn-SOD), and glutathione peroxidase (GSH-Px). These oxidative stress markers were significantly improved in the MnD + MnCl_2_ group, though not fully restored to control levels.

### 3.3. Effects of Mn Deficiency on Lipid Metabolism in the Mice

**Figure 3:**
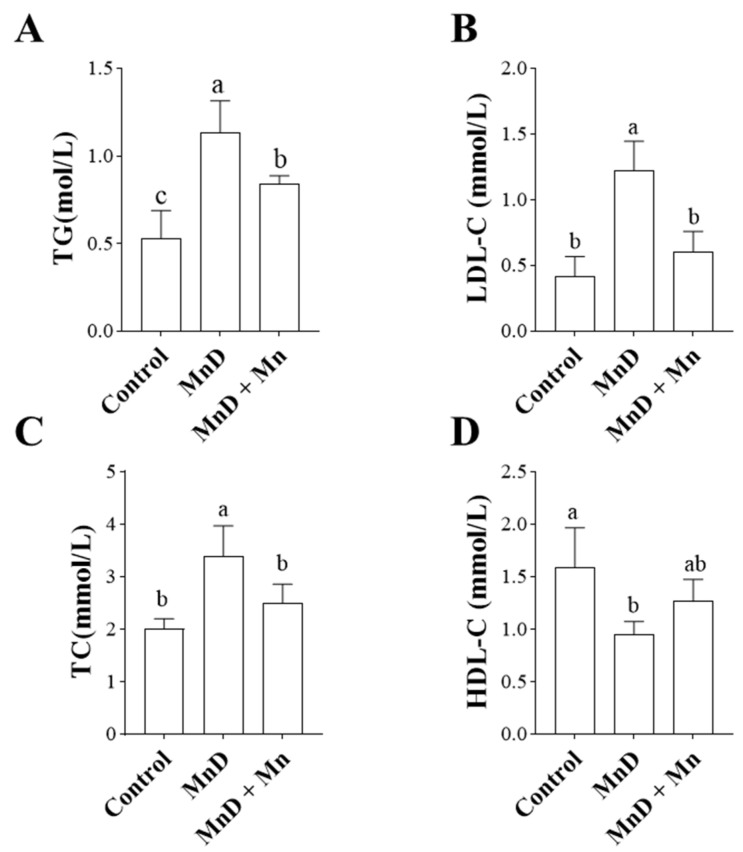
Effects of Mn deficiency on lipid metabolism in the mice. (**A**) TG; (**B**) LDL-C; (**C**) TC; (**D**) HDL-C; data are presented with the mean  ±  standard deviation (n  =  6). ^abc^ Different letters represent significant difference (*p*  <  0.05) within the column, and the same letters represent no significant difference (*p*  >  0.05).

Figure 3 illustrates the effects of Mn deficiency and MnCl_2_ supplementation on lipid metabolism indices in mice. Triglycerides (TG), low-density lipoprotein cholesterol (LDL-C), total cholesterol (TC), and high-density lipoprotein cholesterol (HDL-C) are key indicators of lipid metabolism, with their levels directly reflecting its equilibrium or dysregulation. Figure 3A–C demonstrate that a Mn-deficient (MnD) diet significantly decreased TG, LDL-C, and TC levels (*p* < 0.05); however, these effects were significantly attenuated by moderate MnCl_2_ supplementation (*p* < 0.05). Figure 3D reveals that HDL-C levels were significantly reduced in the MnD group compared to controls (*p* < 0.05), whereas MnCl_2_ supplementation had no significant moderating effect (*p* > 0.05).

### 3.4. Effects of Mn Deficiency on Serum and Hepatic Inflammatory Cytokines in Mice

**Figure 4:**
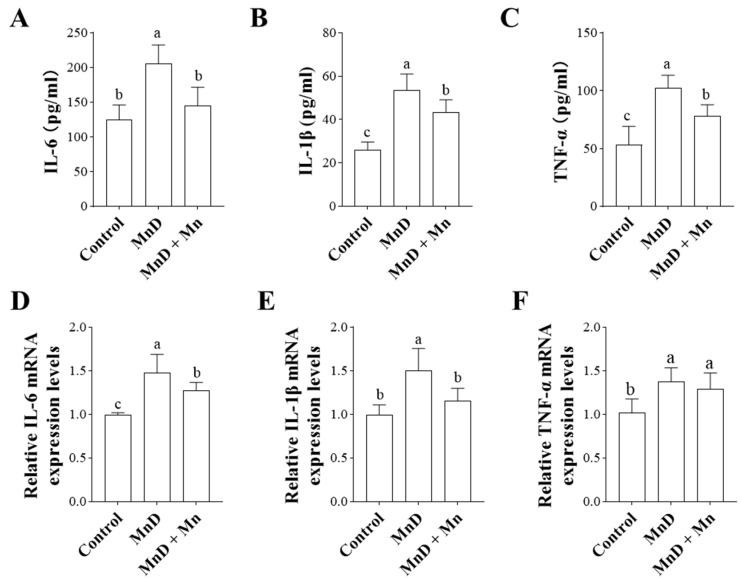
Effects of Mn deficiency on serum and hepatic inflammatory cytokines in mice. (**A**–**C**) The expression level changes in serum levels of IL-6, IL-1β and TNF-α in mice; (**D**–**F**) represent the mRNA relative expression levels of IL-6, IL-1β, and TNF-α, in the liver of mice. Data are presented with the mean  ±  standard deviation (n  =  6). ^abc^ Different letters represent significant difference (*p*  <  0.05) within the column, and the same letters represent no significant difference (*p*  >  0.05).

The effects of Mn deficiency and Mn chloride supplementation on inflammatory factor levels in mouse peripheral blood and liver are shown in Figure 4A–E. Figure 4A–C demonstrates that serum levels of the pro-inflammatory cytokines IL-6, IL-1β, and TNF-α were significantly elevated in the Mn-deficient (MnD) group compared to the control group (*p* < 0.05). Following Mn chloride supplementation, the levels of all three cytokines decreased significantly relative to the MnD group (*p* < 0.05), with IL-6 returning to baseline levels. Additionally, we assessed the mRNA expression of these inflammatory factors in the liver (Figure 4D,E), where the results aligned with the serum findings. Mn deficiency led to a significant upregulation of hepatic pro-inflammatory factors, all of which—except TNF-α—exhibited a marked reduction after Mn supplementation compared to the MnD group.

### 3.5. Transcriptome Analysis of the Effects of Mn Deficiency on the Mouse Liver

**Figure 5:**
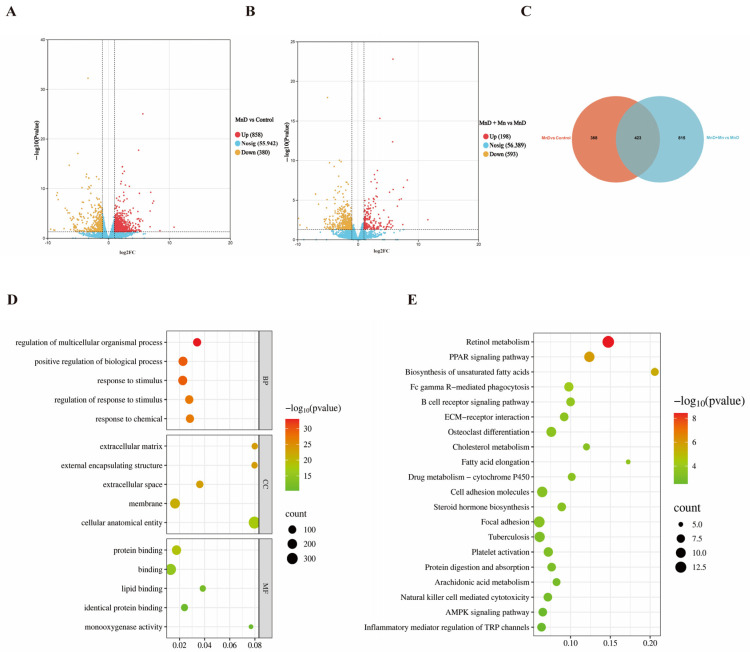
Transcriptome analysis of the effects of mn deficiency on the mouse liver. (**A**) Volcano plots were used to illustrate the changes in gene expression between the MnD group to that of the control group; (**B**) volcano plots were used to illustrate the changes in gene expression between the MnD + Mn group to that of the MnD group; (**C**) a Venn diagram was used to display the overlapping differentially expressed genes between the MnD vs. control and MnD + Mn vs. MnD comparisons; (**D**) the bubble plot displays the top 15 enriched GO terms from the overlapping differentially expressed genes; (**E**) The bubble chart displays the top 20 enriched KEGG pathways from the overlapping differentially expressed genes.

Transcriptomic analysis was conducted to investigate the molecular mechanisms underlying Mn deficiency-induced hepatic oxidative stress, lipid metabolism dysregulation, and inflammatory responses. Compared to the control group, the Mn-deficient (MnD) group exhibited 858 upregulated and 380 downregulated genes (*p* < 0.05) (Figure 5A). Compared to the MnD group, the MnD + MnCL2 group exhibited 198 upregulated and 593 downregulated genes (*p* < 0.05) (Figure 5B). To elucidate Mn-specific regulatory roles, we identified 423 overlapping differentially expressed genes (DEGs) by comparing DEGs from the MnD vs. control and MnD + MnCl_2_ vs. MnD groups (Figure 5C). Functional enrichment analysis of these shared DEGs was performed across biological processes (BP), cellular components (CC), and molecular functions (MF). The top 15 enriched terms (Figure 5D) revealed that BP-associated DEGs were primarily involved in multicellular biological process regulation, positive regulation of biological processes, and response to stimuli. In CC, DEGs were enriched in extracellular matrix, external encapsulated structures, and extracellular space, while MF-associated DEGs were predominantly related to protein binding, binding, and lipid binding. Additionally, KEGG pathway analysis highlighted the top 20 enriched pathways (Figure 5E), with shared DEGs significantly associated with retinol metabolism, PPAR signaling, and biosynthesis of unsaturated fatty acids.

### 3.6. Effects of Mn Deficiency on Molecules Related to the PPAR Signaling Pathway in the Liver

**Figure 6:**
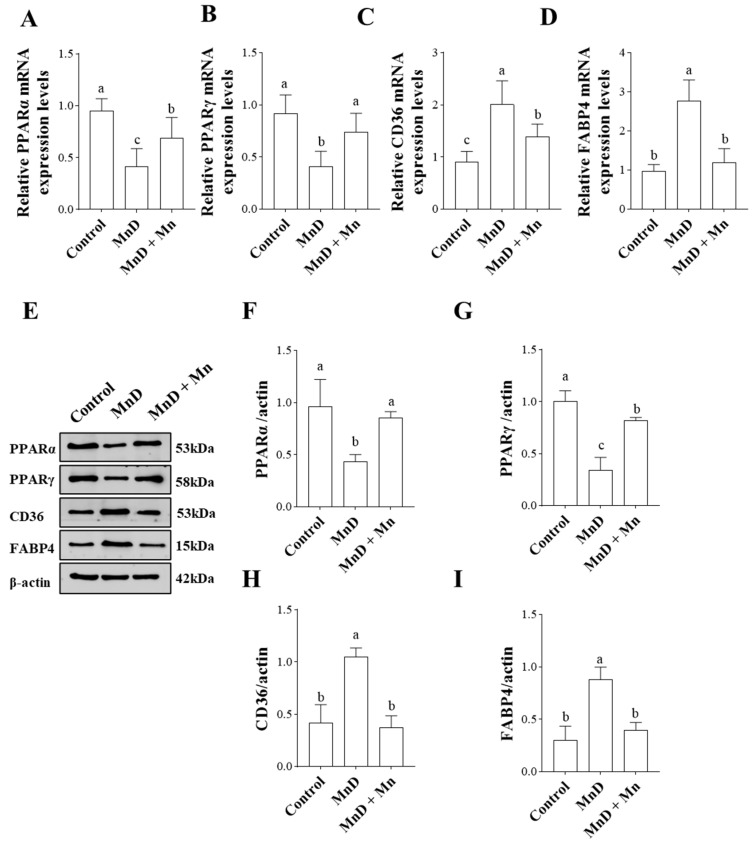
Effects of Mn Deficiency on Molecules Related to the PPAR Signaling Pathway in the liver. (**A**–**D**) The mRNA expression of PPAR pathway related molecules (relative to β-actin); (**E**) The representative band pictures of PPAR pathway related molecules; (**F**–**I**) Relative protein expression of PPAR pathway related molecules (relative to β-actin). Notes: Data are presented with the mean ± standard deviation (n = 6). ^abc^ Different letters represent significant difference (*p* < 0.05) within the column, and the same letters represent no significant difference (*p* > 0.05).

Based on transcriptome KEGG analysis, differentially expressed genes (DEGs) were significantly enriched in the PPAR signaling pathway, which plays a crucial role in oxidative stress, lipid metabolism, and inflammatory responses. This study evaluated the expression of key PPAR pathway proteins (PPARα, PPARγ, CD36, and FABP4) at both the protein and gene levels.As shown in Figure 6A–D, Mn deficiency (MnD) significantly downregulated hepatic mRNA expression of PPARα and PPARγ compared to the control group (*p* < 0.05), while upregulating CD36 and FABP4 expression (*p* < 0.05). In contrast, the MnD + MnCl_2_ group exhibited significantly higher PPARα and PPARγ mRNA levels than the MnD group (*p* < 0.05), along with significantly lower CD36 and FABP4 expression (*p* < 0.05).

Furthermore, we investigated the impact of Mn deficiency on PPAR signaling pathway key proteins (Figure 6E–I). The MnD group showed significantly reduced protein expression of PPARα and PPARγ compared to the control (*p* < 0.05), whereas the MnCl_2_ group exhibited higher expression than the MnD group (*p* < 0.05). Conversely, CD36 and FABP4 protein levels followed an opposite trend.

## 4. Discussion

Mn is an essential trace element critical for normal growth and development in living organisms [33]. It plays a pivotal role in key physiological processes, including antioxidant defense, immune function, and lipid metabolism [34,35,36]. Consequently, Mn deficiency can impair these vital functions. The liver, as the primary site of Mn metabolism and the central metabolic organ, is particularly susceptible to such disruptions. This study investigates the effects of Mn deficiency on hepatic oxidative stress, lipid metabolism, and immune response, aimed at elucidate the underlying mechanisms.

In this study, a Mn deficiency model was established by feeding mice a Mn-deficient diet to investigate its effects on the liver. The results demonstrated that the Mn content in the liver of the Mn-deficient (MnD) group was significantly lower than that of the control group. However, after Mn supplementation, hepatic Mn levels recovered to a level comparable to the control group. The organ coefficient, an important indicator of organ growth and development in life science research, revealed a significant increase in the liver index of the MnD group, suggesting that Mn deficiency inhibits hepatic development in mice. Furthermore, histopathological analysis of MnD group livers showed disorganized hepatic cords, extensive hepatocyte steatosis, and necrosis. Since the normal function of tissues and organs depends on their structural integrity, Mn deficiency likely impairs liver development by disrupting its architecture, which may explain the elevated liver index observed in MnD mice. Additionally, this study evaluated recognized biomarkers of liver function, including ALT, AST, ALB, and ALP [37]. The results indicated that Mn deficiency caused a sharp increase in serum AST, ALT, and ALP levels, along with a significant decrease in ALB, further confirming liver damage. These findings align with previous research by Wang et al. [38], who reported that low dietary Mn intake reduced hepatic and cardiac Mn levels in mice, inducing hydropic degeneration and mild steatosis, ultimately leading to liver injury.

Mn is an essential component of antioxidant enzymes; thus, Mn deficiency may compromise antioxidant function. Oxidative stress—a pathological imbalance between antioxidants and ROS—is a key contributor to multi-organ damage. In this study, we evaluated the effects of Mn deficiency on hepatic oxidative stress by measuring ROS levels and other classical oxidative stress markers. The results demonstrated that Mn-deficient livers exhibited significantly elevated ROS and MDA levels compared to the control group, indicating that Mn deficiency induces oxidative damage in the liver. Concurrently, antioxidant levels, including Mn-SOD, GSH-Px, and T-AOC activity, were significantly reduced. Choi et al. reported that Mn deficiency impaired Mn-SOD activity and exacerbated oxidative stress in a dextran sulfate sodium (DSS)-induced murine colitis model [13]. A similar phenomenon was observed in rats, where Mn deficiency led to decreased hepatic Mn-SOD expression. Additionally, Dong’s study demonstrated that Mn deficiency induces tibial hypoplasia in chickens via the Nrf2 signaling pathway, further supporting our findings that Mn deficiency promotes oxidative stress in vivo [39].

As the metabolic hub of the body, the liver is particularly vulnerable to oxidative stress. The accumulation of oxidative stress may disrupt lipid metabolism in hepatocytes and trigger inflammatory responses. Accordingly, we analyzed key lipid metabolism biomarkers and found that Mn deficiency significantly increased serum levels of triglycerides (TG), total cholesterol (TC), and low-density lipoprotein cholesterol (LDL-C) in mice. Histopathological analysis revealed substantial hepatic steatosis, confirming that Mn deficiency disrupts hepatic lipid metabolism and promotes fat deposition in the liver—a finding consistent with prior studies. Furthermore, we assessed the expression of hepatic inflammatory factors and observed that Mn deficiency significantly upregulated pro-inflammatory cytokines, including IL-6, IL-1β, and TNF-α. These data strongly suggest that Mn deficiency can provoke systemic inflammatory responses, which is consistent with the findings reported by Choi et al. regarding the exacerbation of intestinal inflammation under Mn-deficient conditions [40]. Oxidative stress exacerbates lipid metabolism disorders and inflammatory responses, which in turn amplify oxidative stress, creating a vicious cycle.

We also investigated the molecular mechanism of Mn deficiency-induced oxidative stress, lipid metabolism disorder and inflammation in the liver by transcriptomics, and transcriptomics analyses showed that Mn deficiency-induced liver injury may be mediated by PPAR signaling pathway, which belongs to the nuclear receptor family (including PPARα, PPARβ/δ, and PPARγ) and regulates gene expression after ligand activation [41]. PPAR belongs to the nuclear receptor family and is a transcription factor that regulates gene expression following ligand activation. PPAR works by regulating genes that encode proteins that control metabolic homeostasis and function in multiple organs, including adipose tissue, liver, and heart [42,43]. This pathway is closely related to oxidative stress, lipid metabolism and immunity. Therefore, we further explored its specific molecular mechanism, and we examined the classical factors of the PPAR signaling pathway, and our results showed that the expression levels of PPARα and PPARγ were significantly reduced in the hepatic bells of Mn-deficient mice, whereas the expression of CD36 and FABP4 was increased. Interestingly, although MnCl_2_ supplementation significantly ameliorated most pathological changes induced by manganese deficiency, not all parameters were fully restored to control levels. This partial recovery suggests that the metabolic and structural damage from prolonged deficiency—particularly hepatic steatosis and chronic oxidative stress—may necessitate a longer recovery period than the one week allotted. Alternatively, the dosage used, though sufficient to correct deficiency, may be suboptimal for reversing all downstream effects. These findings point to either a potentially irreversible component of Mn deficiency injury or the involvement of complex adaptive mechanisms not readily reversed by Mn repletion alone. This underscores the severity of deficiency and highlights the importance of timely and adequate Mn intake to prevent persistent hepatic dysfunction. Future studies should explore longer supplementation periods or adjusted dosing regimens to optimize functional recovery.

Although this study was conducted in a mouse model, the findings may hold significant implications for human health. The triad of hepatic oxidative stress, lipid dysregulation, and inflammation in Mn-deficient mice reflects key pathological features of human non-alcoholic fatty liver disease (NAFLD/MASLD) [28]. Epidemiological studies have linked altered Mn status to liver dysfunction in humans, supporting the potential clinical relevance of our mechanistic data [19,20]. These observations suggest that Mn status assessment could serve as a supplementary biomarker for metabolic liver disease risk. Moreover, our results identify the PPAR signaling pathway as a critical mechanistic node, revealing promising therapeutic avenues. Strategies such as enhancing Mn-dependent antioxidant defenses (e.g., using Mn-based complexes) or pharmacologically targeting PPARs (e.g., with fenofibrate or pioglitazone) may help ameliorate liver injury associated with functional Mn deficiency. Future studies should validate these mechanisms in human cohorts and preclinical NAFLD models to evaluate the therapeutic potential of Mn-focused interventions.

This study has several limitations that warrant consideration. The use of young mice may constrain extrapolation to adult or human contexts, owing to metabolic and developmental differences between age groups. The four-week deficiency period followed by one week of supplementation may not adequately reflect the effects of chronic deficiency or the long-term efficacy of replenishment. Moreover, while intraperitoneal administration of manganese allows for precise dosing, it bypasses natural absorption pathways and alters pharmacokinetic profiles compared to oral dietary intake. The dietary deficiency model employed here may also not fully represent other etiologies of manganese dysregulation. Finally, inherent species differences suggest that direct clinical translation should be approached with caution. Thus, our findings are most appropriately interpreted as illuminating fundamental molecular pathways, the relevance of which to human disease necessitates further investigation.

## 5. Conclusions

Our findings suggest a potential pathogenic cascade in which manganese deficiency may initially induce hepatic oxidative stress, potentially leading to suppression of the PPAR signaling pathway. This inhibition of PPARα/γ could subsequently orchestrate downstream manifestations of aberrant lipid metabolism and inflammatory responses. Thus, the PPAR signaling pathway is proposed as a plausible central hub translating oxidative damage into metabolic and inflammatory dysfunction in the manganese-deficient liver.

## Figures and Tables

**Table 1:** The contents of manganese-deficient diet.

Formula	g/Kg
Casein, low Cu & Fe	200.0
DL-Methionine	3.0
Sucrose	549.99
Corn starch	150
Corn oil	50
Mineral mix, Mn-deficient (81062)	35
Vitamin mix, AIN-76A (40077)	10
Choline bitartrate	2.0
Ethoxyquin, antioxidant	0.01

**Table 2:** The sequence of primers used in the study.

Gene	Forward (5′ → 3′)	Reverse (3′ → 5′)
*IL-6*	TAGTCCTTCCTACCCCAATTTCC	TTGGTCCTTAGCCACTCCTTC
*IL-1β*	GCAACTGTTCCTGAACTCAACT	ATCTTTTGGGGTCCGTCAACT
*TNF-α*	CCTGTAGCCCACGTCGTAG	GGGAGTAGACAAGGTACAACCC
*PPARα*	GAAGCCTACCTGAAGAAC	CGGATTGTTGCTAGTCTT
*PPARγ*	CATCAGGCTTCCACTATG	GACAGTTAAGATCACACCTAT
*CD36*	AGCACTGAAGAATCTGAAG	AACATCACTACTCCAACAC
*FABP4*	GATCATCAGCGTAGAAGG	TTCACTTTCCTGTCATCTG
*β-actin*	GGCTGTATTCCCCTCCATCG	CCAGTTGGTAACAATGCCATGT

## Data Availability

The article outlines the study’s original contributions, and the corresponding author can address further queries. The data are not publicly available due to privacy restrictions.
